# A double blind, randomised placebo controlled trial of topical 2% viscous lidocaine in improving oral intake in children with painful infectious mouth conditions

**DOI:** 10.1186/1471-2431-11-106

**Published:** 2011-11-21

**Authors:** Sandy M Hopper, Franz E Babl, Michelle McCarthy, Chasari Tancharoen, Katherine J Lee, Ed Oakley

**Affiliations:** 1 Department of Emergency Medicine Royal Children's Hospital, Flemington Rd, Parkville, Victoria 3052, Australia; 2 Murdoch Children's Research Institute Flemington Rd Parkville Victoria 3052 Australia; 3 Department of Paediatrics, Faculty of Medicine, Dentistry and Health Sciences, University of Melbourne, Victoria 3010 Australia; 4 Southern Clinical School, Faculty of Medicine, Nursing and Health Sciences, Monash University, Victoria 3800 Australia; 5 Department of Emergency Medicine Monash Medical Centre, Clayton Rd, Clayton Victoria 3168 Australia

## Abstract

**Background:**

Painful infectious mouth conditions are a common presentation to emergency departments. Although self limiting, painful ulcerative lesions and inflamed mucosa can decrease oral intake and can lead to dehydration. Oral analgesia is of limited efficacy and is often refused by the patient. Despite widespread use of oral 2% viscous lidocaine for many years, there is little evidence for its efficacy as an analgesic and in aiding oral intake in children with painful infectious mouth conditions. This study aims to establish the effectiveness of 2% viscous lidocaine in increasing oral intake in these children by comparing it with placebo.

**Methods/Design:**

This study is a randomised double-blind placebo controlled trial of children between 6 months and 8 years of age with painful infectious mouth conditions defined as gingivostomatitis (herpetic or non herpetic), ulcerative pharyngitis, herpangina and hand foot and mouth disease as assessed by the treating clinician in association with a history of poor oral fluid intake. It will be conducted at a single tertiary paediatric emergency department in Melbourne Australia.

20 patients have already been randomised to receive 2% lidocaine or placebo in a pilot study to determine the sample size in a preplanned adaptive design. A further 80 patients will be randomised to receive either 2% lidocaine or placebo. The placebo agent is identical to lidocaine in terms of appearance, flavour and smell. All clinical and research staff involved, patients and their parents will be blinded to treatment allocation.

The primary endpoint is the amount of fluid ingested by each child, expressed in ml/kg, within 60 minutes from the time of administration of the study mixture. Secondary endpoints are the proportion of patients ingesting 5 ml/kg and 10 ml/kg at 30 and 60 minutes after drug administration and the incidence of adverse events. Longer term outcomes will include the proportion of patients requiring hospital admission and length of emergency department stay.

**Discussion:**

This trial will define the role of 2% lidocaine in the treatment of painful infectious mouth conditions

**Trial registration:**

The trial is registered with the Australian and New Zealand Clinical Trials Registry - ACTRN12609000566235.

## Background

Painful infectious mouth conditions are a common presentation to paediatric emergency departments. These are usually viral conditions such as gingivostomatitis (herpetic and non herpetic), ulcerative pharyngitis, herpangina and hand foot and mouth disease. Most of the paediatric literature concerns primary herpetic gingivostomatitis (PHGS) caused by infection with the herpes simplex virus. Although self limiting, there is considerable morbidity from decreased oral intake. Amir et al found that 89% of patients with PHGS drank less than normal [1]. The oral lesions can persist for 7 to 18 days with the risk of dehydration [2]. The presence of extra-oral lesions in addition to intra-oral lesions is highly indicative of PHGS [3], and may be used to differentiate PHGS from other ulcerative mouth conditions of children: mainly herpangina and hand foot and mouth disease (both caused by the coxsackie virus). In herpangina, lesions are typically seen on the posterior portion of the oral cavity and palate, with an absence of extra-oral lesions. Hand foot and mouth disease typically has both intra-oral and extra-oral lesions and the distribution of characteristic lesions on the hands and feet distinguishes it from PHGS [4]. These findings are particularly valuable as the majority of PHGS cases are diagnosed on clinical findings alone. Treatment for such conditions is generally symptomatic and therefore a precise diagnosis is not critical as such.

The mainstay of care for children with gingivostomatitis is supportive and expectant. The primary goal of therapy in acute care is directed towards pain relief from ulcerative lesions and inflamed mucosa, thus facilitating oral fluid intake to prevent dehydration. Often, routine oral analgesia is the first mode of therapy attempted by parents at home. However, it may have little effect in the relief of pain and is often refused by the patient. If oral intake cannot be improved, there is a subsequent risk of admission to hospital for intravenous (IV) or nasogastric (NG) fluids. Amir et al [3] found that 3 out of 36 (8%) children presenting with herpes gingivostomatitis experienced dehydration and required hospitalisation for IV rehydration.

Treatment trials for painful infectious mouth conditions have focussed on the efficacy of acyclovir and similar antiviral agents for treating PHGS with controversy about the strength and weakness of the evidence of its efficacy [2,5,6]. Regardless, acyclovir use is of limited relevance where acute fluid intake of a child in the ED is concerned and the exact diagnosis is often not known. Many agents have been described for painful ulcerative mouth conditions in adults and children including gingivostomatitis, oral candidiasis and aphthous ulcers. Agents used for one or a number of conditions include viscous lidocaine, benzocaine preparations, diphenhydramine elixir, coating agents including Maalox, milk of magnesia and Kaopectate, antibiotics particularly chlorhexidine and tetracycline and topical steroids [7-21]. In a retrospective chart review of 48 non immune suppressed paediatric patients presenting with PHGS at the Children's Hospital in Buffalo, United States[2], all were treated with analgesics such as acetaminophen or ibuprofen, 35 were treated with a mixture of Maalox and diphenhydramine, 8 with acyclovir, 7 with viscous lidocaine and 11 received two or more of these therapies. A major textbook of Paediatric Emergency Medicine [10] mentions rinsing with viscous lidocaine, as well as "magic mouthwash" (a mixture of Kaopectate and Benadryl) as treatment options. 2% viscous lidocaine has been recommended in the study hospital ED Clinical Practice Guidelines (CPG) for the treatment of herpes gingivostomatitis for many years [9]. To date, however, there have been no RCTs investigating any topical anaesthetic for painful ulcerative conditions of the mouth in previously health children.

There are some data from adult ulcerative mouth conditions and patients with cancer related mucositis. In a double blind RCT Saxen et al [11] randomised 60 adults with aphthous ulcers to one of 3 treatment groups - 3% diclofenac in 2.5% hyaluronan, 2.5% hyaluronan or 3% viscous lidocaine. For spontaneous and stimulated pain a significant reduction in pain was shown in all 3 groups (p < 0.01) with no demonstrable difference between the 3 topical agents. Akhionbare and Ojehanon [12] randomised 30 patients with aphthous ulcers to either 2% plain lidocaine or 2% lidocaine with adrenaline. 87% of patients experienced a complete relief of pain after application of either solution. Although different etiologically, oral mucositis often results in unrelenting pain causing an inability to eat and drink with subsequent risk of malnutrition and dehydration. In this respect it is similar to severe infectious mouth conditions. Yet, a review of the literature on the management of mucositis demonstrates a lack of consensus of scientific opinion on optimal treatment for relieving painful mucositis [22] and the use of a large variety of different topical agents including diphenhydramine, viscous lidocaine, magnesium hydroxide and aluminium hydroxide (Maalox), nystatin and corticosteroids has been described [23]. RCT data are limited and a Cochrane review [24] found that there was "no proven and satisfactory treatment available" for oral mucositis in the adult population. Similarly, a paediatric review [22] revealed a paucity of controlled trials in children, highlighting that current practice is governed more by clinical experience rather than scientific evidence.

Lidocaine, the first amide-type local anesthetic, is commonly used topically for a number of indications. A flavoured 2% solution of lidocaine hydrochloride (2% viscous lidocaine, trade name Xylocaine Viscous^® ^Astra Zeneca) is commercially available. Local anaesthesia is achieved within approximately 5 minutes and the duration of anaesthesia lasts between 20 and 30 minutes [25]. The recommended maximum dose of lignocaine is 3 mg/kg, 3 hourly, which equals 0.15 ml/kg of 2% viscous lidocaine. Adverse events from topical lidocaine administration are uncommon and have only been reported when used beyond the recommended doses [26,27].

Ulcerative lesions and inflamed mucosa due to viral infections in children are frequent reasons for presentations and can be associated with decreased oral intake and the risk of dehydration. Despite their widespread use, at this time there are no data to support the use of topical agents, in particular topical lidocaine for painful infectious mouth conditions in otherwise healthy children. We set out to assess if 2% viscous lidocaine can increase oral intake in these children by comparing it with placebo.

## Methods/Design

### Study Aims

The primary research question of this study is whether topical 2% viscous lidocaine is effective in improving the poor oral intake associated with painful infectious mouth conditions compared to placebo in children within 60 minutes of administration.

### Study Design and Setting

This study is a randomised double-blind placebo controlled trial. It will be conducted at a single tertiary paediatric emergency department (ED) in the Royal Children's Hospital (RCH), Melbourne, Australia.

### Ethical Considerations

The study has ethical approval at the study hospital. All parents/guardians are provided with both verbal and written information about the study and written informed consent is obtained prior to enrolment of their child into the trial.

### Subject Selection

Children satisfying the inclusion and exclusion criteria who present to the recruiting hospital ED during the study period are eligible for enrolment.

### Definition of Disease State

The eligible participants will be patients aged 6 months to 8 years, who present with gingivostomatitis (herpetic or non herpetic), ulcerative pharyngitis, herpangina and hand foot and mouth disease as assessed by the treating clinician, in associated with a history of poor oral fluid intake (as assessed by parent and defined as oral fluid intake of less than 10 ml/kg of fluid in the preceding 2 hours).

### Inclusion and Exclusion Criteria

Inclusion and exclusion criteria are presented in Table 1. Exclusion criteria include hypersensitivity to any of the contents of the study medication or similar drugs (other amide local anaesthetics), and patients with diseases (such as epilepsy impaired cardiac conduction, bradycardia, impaired hepatic function) that may be complicated by the study drugs.

**Table 1:** Inclusion and exclusion criteria

**Inclusion criteria - all of:**
Diagnosed by the treating doctor with ulcerative pharyngitis, herpangina, hand foot and mouth disease, herpetic gingivostomatitis OR non-herpetic gingivostomatitis
Parental complaint of child's poor oral intake
Intake of less than 10 ml/kg of fluid within 2 hours preceding presentation to RCH ED

**Exclusion criteria - any of:**
Presence of more than 2 vomits within 24 hours preceding presentation to RCH ED
Presence of active painful dental disease (caries, dental abscess) or painful recent mouth trauma, mouth burn, or post-operative state (minimum of 5 days)
Systemic toxicity related to infection, as defined by the treating doctor
Severe dehydration requiring immediate therapy, as defined by the treating doctor
Known allergy to local anaesthetic, gelatine, methylcellulose, cherry flavouring, paracetamol or ibuprofen
Chronic renal or liver impairment
History of epilepsy or cardiac disease
Presence of acute porphyria
Presence of malignancy
Current use of anti-arrhythmic drugs, xylocaine, phenytoin, cimetidine or beta-blockers, warfarin, lithium, angiotensin converting enzyme (ACE) inhibitors, thiazide diuretics, frusemide, aspirin, salicylates, probenecid, anti-diabetic medications, zidovudine, cardiac glycosides or methotrexate
More than 1 dose of 2% viscous lignocaine or medications containing lignocaine as the active ingredient for this episode of illness
Pre-existing upper airway obstruction and/or swallowing difficulties
Analgesia taken within 1 hour preceding enrolment to study Non-English speaking parents/guardians

RCH ED Royal Children's Hospital emergency department

### Patient Randomisation

Patients who fulfil the eligibility criteria and whose parents provide informed consent will be randomised to either 2% viscous lidocaine or placebo.

The randomisation schedule will be computer generated using block randomisation by the Clinical Biostatistics and Epidemiology Unit (CEBU, Murdoch Children's Research Institute, Melbourne, Australia). Random block sizes will be used. The randomisation will not be stratified. Sequentially numbered bottles of either lidocaine or placebo will be arranged according to this schedule by a study pharmacist. Once a patient has been randomised they will be given the next available study number which will equate to an allocation of study drug dispensed in two bottles (labelled Bottle A and Bottle B).

At the time of randomisation patients will be given a dose of study solution from Bottle A which will contain the study drug according to their random allocation. Bottle B will contain the alternative mixture i.e. lidocaine if randomised to placebo and placebo if randomised to lidocaine. After 60 minutes the treating clinician may prescribe a dose from this second bottle if clinically warranted. This ensures that all participants in the study have access to the active treatment.

All study drugs will be packaged in blinded containers stored in a locked cupboard labelled only with the study details and the study number. Only the study pharmacist will be aware of the treatment allocation.

### Randomised treatments

#### Study drug: 2% viscous lidocaine mixture (Xylocaine Viscous^®^, Astra Zeneca)

This is a viscous mixture of 2% lidocaine hydrochloride, made up with methyl hydroxybenzoate, propyl hydroxybenzoate, sodium hydroxide, saccharin sodium, cherry extract (water, flavour, citric acid and amaranth), carmellose sodium and water for injections. The dose of 2% viscous lidocaine at the RCH conforms to the local Paediatric Pharmacopoeia at 0.15 ml/kg [28].

#### Placebo: contains methylcellulose and cherry flavouring

The placebo will be made up by the RCH Pharmacy from stock ingredients. The placebo will be identical to the study drug in terms of appearance, flavour and smell.

### For both groups

0.15 ml/kg of lidocaine/placebo will be administered once at the time of randomisation by the treating nurse via a measured syringe to the oral cavity of the participant ensuring coverage to inflamed mucosa and ulcerative lesions. If the participant is mature enough, he/she will be instructed to gargle and spit out the study drug/placebo. If the participant is not mature enough to follow these instructions, he/she may swallow or spit out the study drug/placebo. It will be recorded if the lidocaine/placebo is spat out within 1-2 seconds of administration. Further lidocaine/placebo will not be administered.

With the exception of the bottles of study drug, 2% viscous lidocaine may not be prescribed by the treating clinician during the 90 minute study period or for the preceding 90 minutes due to risk of lidocaine toxicity.

### Breaking of the Study Blind

#### On Study

In a medical emergency, when management of a participant's condition requires knowledge of the investigational product, the randomisation code may be broken to determine the treatment allocation of the participant. If this is required the investigator can contact the pharmacy to obtain the treatment identity. If possible, such emergencies will be discussed with the Principal Investigator before disclosure of the treatment allocation (or as soon as possible thereafter). Reasons for breaking a code must be clearly explained and justified on the participant's CRF. The date on which the code is broken together with the identity of the person(s) responsible for requesting the code to be broken will also be documented.

#### Following Completion of the Study

Study unblinding will only take place once the Statistical Analysis Plan has been agreed by the trial team and after the final database has been locked. This will be achieved by obtaining the schedule linking Randomisation Numbers with the administered treatment from CEBU.

### Study Procedure

#### Dose and administration schedule

**Figure 1:**
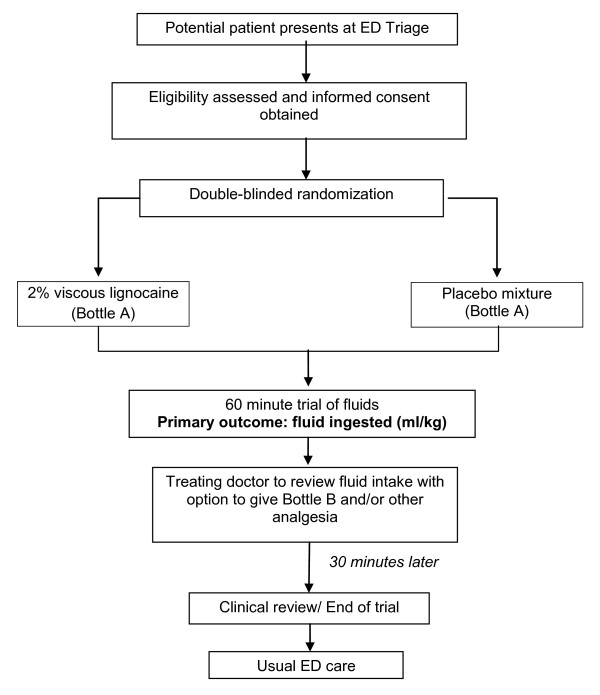
**Study Flow Chart**. The schedule of study observations that will be recorded during the 60 minute study period and the 30 minute observation period. (legend) ED emergency department

The single dose of study drug will be administered at time 0 as outlined in Study Flow Chart (Figure 1). The amount of fluid ingested within 30 and 60 minutes will be recorded on a Fluid Balance Chart.

After the end of the 60 minute fluid trial and once the outcome data has been collected, the treating clinician will complete a fluid assessment on the participant. At this point, if the treating clinician feels that a participant has not had enough fluid, he/she may decide to:

• Administer the second bottle allocated to that participant (Bottle B) - participants previously given placebo will receive the active drug (i.e. 2% viscous lidocaine), and vice versa, and/or

• Administer other oral analgesia e.g. paracetamol or ibuprofen

The study will terminate at 90 minutes (i.e. 30 minutes after the end of the 60 minute fluid trial for assessment of adverse events) where the participant will be reviewed for signs of aspiration. However, if at any point during the trial the participant starts coughing and/or show other signs of aspiration, he/she will be urgently reviewed by the treating clinician. A completed participant is one who has had the solution administered, and has had their fluid intake monitored for a minimum of 60 minutes from the time of administration of the study mixture. If the participant does not complete the 60 minute study period, the reason for the premature termination will be documented on the CRF. After the completion of the 60 minute trial of fluids routine care will follow as directed by the treating clinician.

Complications recorded will include: allergic reactions (including anaphylaxis), seizures, cardiac arrhythmia, and clinical episodes of aspiration.

### Outcome measures

*The primary outcome *is the amount of fluid ingested by each child, expressed in ml/kg, within 60 minutes from the time of administration of the study mixture.

### *The secondary outcome *measures are in 2 groups

#### Short term

those within 60 minutes - and longer term - those between 60 and 90 minutes. Comparisons between groups will only be made in the short term outcomes since both groups will have access to lidoocaine after 60 minutes.

#### Short term

(1) the proportion of subjects in each arm to have ingested more than 5 ml/kg of fluid within 30 and 60 minutes from the time of administration of the study mixture; (2) the proportion of subjects in each arm to have ingested more than 10 ml/kg of fluid within 30 and 60 minutes from the time of administration of the study mixture.

#### Longer term

(1) incidence of adverse events (seizures, cardiac arrhythmias and aspiration) within the 90 minute study period; (2) the proportion of subjects who require fluid administration via intravenous line or nasogastric tube (oral hydration failures); (3) the proportion of subjects requiring hospital admission; (4) duration of stay in the ED

### Sample size, power and statistical methods

There is currently no published literature on the effectiveness of 2% viscous lidocaine for improving oral fluid intake in painful infectious mouth conditions, hence we carried out a pilot study of 20 patients to document the mean fluid intake and spread of the data.

The data from the pilot study found a mean difference of 4.3 mls/kg (standard deviation = 7 mls/kg) ingested over a 60 minute period between the two treatment groups. Assuming a slightly conservative estimate of the treatment effect of 4 mls/kg (standard deviation = 7), would require a sample size of 50 children per group (100 children in total), based on 80% power and 5% significance. A review of expert clinical opinion (10 paediatric emergency attendings in Melbourne, Australia) concluded that a difference of 4-5 mls/kg represented a clinically important difference.

Based on the recruitment rate in the pilot study (20 participants in 5 months) we expect patient recruitment to take 2 years (the 20 patients from the pilot study will be included in the final analysis). We have not allowed for any loss-to-follow up in this time line as we expect to retain all patients for the 60 minutes needed for the study.

Analysis will be by intention to treat. Data will be analysed descriptively and statistically using the STATA data analysis program. Data for the primary outcome will be reported with an estimate of the difference in means and its 95% confidence interval - obtained using an unadjusted linear regression. Since this is an RCT, and the outcome measure is measured on a continuous scale, the t-test will be used to compare the 2 groups. If necessary due to skewed data, the data will be log-transformed. Secondary outcomes in the short term group will be analysed with t-tests for continuous data and chi-squared tests for categorical data, while the longer term group will have descriptive statistics only. As a sensitivity analysis, all treatment comparisons for primary and secondary outcomes will also be presented adjusted for age at presentation to account for any chance imbalance between the treatment groups with respect to this potentially confounding factor using linear and logistic regression models for continuous and binary outcomes respectively.

### Adverse experiences

All adverse experiences either observed by the investigator or one of the clinical staff, or reported by the patient's parents/guardians spontaneously or in response to a direct question, that occur during the 60 minute study period, and the 30 minute observation period, will be evaluated by the investigator and noted in the adverse experience section of the patient's CRF. Events after the study period also thought to be due to a study intervention will be included.

### Serious adverse events

A serious adverse event (SAE) is generally defined as any event that is fatal, life-threatening, permanently disabling, incapacitating or results in hospitalisation, prolongs a hospital stay or is associated with congenital abnormality, carcinoma or overdose. SAEs will be judged as to how likely they are to be related to the study drug; this judgement is to be made by the participant's treating clinician. These may include conditions such as pulmonary aspiration, seizures and cardiac arrhythmia.

### Reporting SAEs

SAEs need to be reported within 24 hrs by telephone to the principal investigator. The SAE will be reported to the ethics committee within 48 hours. SAEs will also be reported to the consultant of the child's treating team. The medical consultant will initiate appropriate management and inform the family if the family is not already aware of the event. This reporting is the responsibility of the principal investigator. All SAEs will be followed by the study investigator until resolution.

### Limitations

This trial has some potential limitations. While the study endeavours to capture all patients presenting with painful infectious mouth conditions, patients will only be approached if researchers are available in the ED. In addition, some patients will mistakenly receive oral analgesia by triage nursing staff at the triage desk or while waiting to be seen even though research staff are available and therefore become ineligible for enrolment.

### Time plan

Recruitment has begun in early 2011 for the 80 remaining patients needed for the study. It is anticipated patient recruitment will be completed by the end of 2013.

## Abbreviations

SAE: serious adverse event.

## Competing interests

The authors declare that they have no competing interests.

## Authors' contributions

SH and FEB were responsible for identifying the research question, study design and research protocol. MMcC, KL and EO have all contributed to the development of the protocol and study design, as members of the research team. EO was responsible for the drafting of this paper. All authors provided comments on the drafts and have read and approved the final version. SH takes responsibility for the manuscript as a whole

## Pre-publication history

The pre-publication history for this paper can be accessed here:

http://www.biomedcentral.com/1471-2431/11/106/prepub
